# Pharmacokinetics and Tissue Distribution of Gingerols and Shogaols from Ginger (*Zingiber officinale* Rosc.) in Rats by UPLC–Q-Exactive–HRMS

**DOI:** 10.3390/molecules24030512

**Published:** 2019-01-31

**Authors:** Ling-Ling Li, Ying Cui, Xing-Han Guo, Kai Ma, Ping Tian, Jing Feng, Jun-Ming Wang

**Affiliations:** 1 School of Pharmacy, Henan University of Chinese Medicine, 156 Jinshui east Road, Zhengzhou 450046, China; openleeling@163.com (L.-L.L.); gxh5508@163.com (X.-H.G.); fj.xyz@163.com (J.F.); mjw98@126.com (J.-M.W.); 2 Collaborative Innovation Center for Respiratory Disease Diagnosis and Treatment & Chinese Medicine Development of Henan Province, 156 Jinshui east Road, Zhengzhou 450046, China; 3 Henan Province Chinese Medicine Research Institute, Zhengzhou 450046, China; zzmk1968@126.com (K.M.); tianping082@163.com (P.T.)

**Keywords:** pharmacokinetics, tissue distribution, ginger, gingerols, shogaols, UPLC–Q-Exactive–HRMS

## Abstract

Gingerols and shogaols are recognized as active ingredients in ginger and exhibit diverse pharmacological activities. The preclinical pharmacokinetics and tissue distribution investigations of gingerols and shogaols in rats remain less explored, especially for the simultaneous analysis of multi-components. In this study, a rapid, sensitive, selective, and reliable method using an Ultra-Performance Liquid Chromatography Q-Exactive High-Resolution Mass Spectrometer (UPLC-Q-Exactive–HRMS) was established and validated for simultaneous determination of eight compounds, including 6-gingerol, 6-shogaol, 8-gingerol, 8-shogaol, 10-gingerol, 10-shogaol, Zingerone, and 6-isodehydrogingenone in plasma and tissues of rats. The analytes were separated on a Syncronis C18 column (100 × 2.1 mm, 1.7 µm) using a gradient elution of acetonitrile and 0.1% formic acid in water at a flow rate of 0.25 mL/min at 30 °C. The method was linear for each ingredient over the investigated range with all correlation coefficients greater than 0.9910. The lowest Lower Limit of quantitation (LLOQ) was 1.0 ng/mL. The intra- and inter-day precisions (Relative Standard Deviation, RSD%) were less than 12.2% and the accuracy (relative error, RE%) ranged from −8.7% to 8.7%. Extraction recovery was 91.4–107.4% and the matrix effect was 86.3–113.4%. The validated method was successfully applied to investigate the pharmacokinetics and tissue distribution of eight components after oral administration of ginger extract to rats. These results provide useful information about the pharmacokinetics and biodistribution of the multi-component bioactive ingredients of ginger in rats and will contribute to clinical practice and the evaluation of the safety of a Chinese herbal medicine.

## 1. Introduction

Ginger, the rhizome of *Zingiber officinale* Rosc. (*Z. officinale*), is frequently utilized as both a spice and a traditional herbal medicine for centuries all over the world due to its health benefits [1,2,3]. Several regulatory authorities identify ginger as a safe supplemental herbal [4,5], and it is also implemented in complementary and alternative medicine preparations for the treatment of different diseases, such as headaches, colds, and fevers, for reliving gingivitis, and as an anti-emetic, expectorant, and appetite stimulant [6,7,8]. Furthermore, previous studies have revealed that ginger possesses anti-tumor activity [9,10].

More than 200 compounds are identified from ginger, and its bioactive components include volatile oils, anthocyanins, tannins, and pungent phenolic compounds known as gingerols, shogaols, and sesquiterpenes [11,12]. Ginger is widely cultivated as a spice for its aromatic and pungent components including essential oil and oleoresins [13]. The non-volatile compounds are gingerols, shogaols, paradols, and zingerone [14], sharing the same parent nucleus structure and are responsible for pharmacological functions in fresh ginger [15,16]. Previous studies demonstrated that gingerols and shogaols have diverse pharmacological activities, especially for 6-gingerol and 6-shogaol, have been proven to exhibit significant anti-hyperglycemic activity [17], antioxidant [18], analgesic [19], anti-inflammatory [20], anti-cancer [21], anti-arteriosclerosis [22], and lifespan-extending functions [23,24].

Owing to the abundant pharmacological actions of gingerols and shogaols, it is better to understand their absorption and distribution in vivo. There are quite a few researches about pharmacokinetic studies of 6-gingerol, 8-gingerol, 10-gingerol and 6-shogaol [25,26,27]; however, fewer components of ginger were investigated and their tissue distribution was not involved. Therefore, it is necessary and meaningful to develop a more accurate and selective bioanalytical method for the determination of more active ingredients in plasma and tissues to understand the characterization and diversity of the pharmacokinetic and tissue distribution properties of ginger.

Ultra-Performance Liquid Chromatography (UPLC) combined with High-Resolution Mass Spectrometry (HRMS) is a powerful technique utilized for diverse applications due to its very high sensitivity and selectivity. In recent years, it has rapidly developed an important method to measure the composition of trace components in food and drugs [28,29]. Therefore, the aim of this study was to develop and validate a method for simultaneous measurement of eight components (6-gingerol, 6-shogaol, 8-gingerol, 8-shogaol, 10-gingerol, 10-shogaol, Zingerone and 6-isodehydrogingenone) in rat plasma and organs using an Ultra-Performance Liquid Chromatography Q-Exactive High-Resolution Mass Spectrometer (UPLC–Q-Exactive–HRMS), and investigate their pharmacokinetics and tissue distribution. To the best of our knowledge, this is the first time a method has been established for simultaneously measuring eight components of fresh ginger in rat plasma and organs and investigating their pharmacokinetics and tissue distribution in rats.

## 2. Results and Discussion

### 2.1. Method Development

Since analytes were present in relatively low concentrations and in a complex matrix environment, it was important to optimize the chromatographic and mass spectrometric conditions to develop a rapid, sensitive, and precise method to circumvent these potentially confounding issues as well as an efficient extraction procedure for all analytes.

#### 2.1.1. Chromatography and Mass Spectrometry

To obtain optimized responses, suitable retention times, and ideal peak shapes for the analytes, the chromatographic condition was optimized through trial and error based on previous studies [26,27]. Methanol, acetonitrile, 0.1% formic acid, and chromatographic grade water were selected as mobile phase candidates. Finally, a gradient elution with acetonitrile and water containing 0.1% formic acid achieved our purpose and was finally adopted as the mobile phase for the chromatographic separation. Additionally, formic acid (0.1%) was added to water to improve peak shape of the analytes, and the ionization efficiency was improved as the acidic mobile phase provided abundant H^+^. Run time of total chromatography analysis was 12 min with retention times of 2.64 min for 6-gingerol, 4.79 min for 6-shogaol, 4.29 min for 8-gingerol, 6.65 min for 8-shogaol, 6.16 min for 10-gingerol, 8.33 min for 10-shogaol, 2.64 min for Zingerone, 5.83 min for 6-isodehydrogingenone and 6.17 min for Atractylenolide I, used as the internal standard (IS).

**Figure 1:**
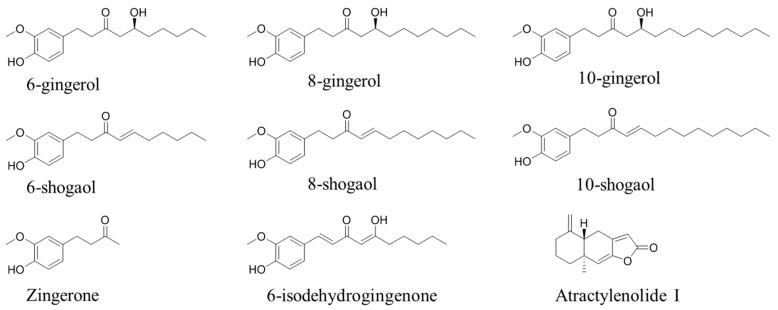
Chemical structures of eight target analytes and Atractylenolide I, the internal standard (IS).

For MS condition, the feasibility of electrospray in positive and negative ion modes was evaluated during assay development. We found that the response observed in positive ion mode was higher than that in negative mode. Full scan mode was used to collect the molecular ion peaks with the mass range of 100–1000. The most abundant ions in the spectra ([M + H]^+^ of 6-, 8-, 10-shogaols, 6-isodehydrogingenone and IS, [M + H − H_2_O]^+^ of 6-, 8-, 10-gingerols and Zingerone) were selected for sensitive quantitation. The *m*/*z* of 6-gingerol and 6-shogaol was 277.18024, 8-gingerol and 8-shogaol was 305.21188, 10-gingerol and 10-shogaol was 333.24222, Zingerone was 177.09105, 6-isodehydrogingenone was 291.15891, and IS was 231.13847. The structures of eight analytes and IS are shown in Figure 1.

#### 2.1.2. Sample Preparation and Internal Standard Selection

An effective and simple protein precipitation and a liquid–liquid extraction method was employed to remove protein and potential interference from bio-samples (plasma and tissue samples) prior to LC–MS analysis. Methanol was used as the protein precipitation solvent and ethyl acetate was applied to extract target analytes.

A suitable internal standard is a key factor in biological sample analysis. Atractylenolide I was chosen to be an internal standard because of its similar retention time to other analytes and lack of endogenous interference in rat plasma and organ samples.

### 2.2. Method Validation

#### 2.2.1. Selectivity and Linearity

**Figure 2:**
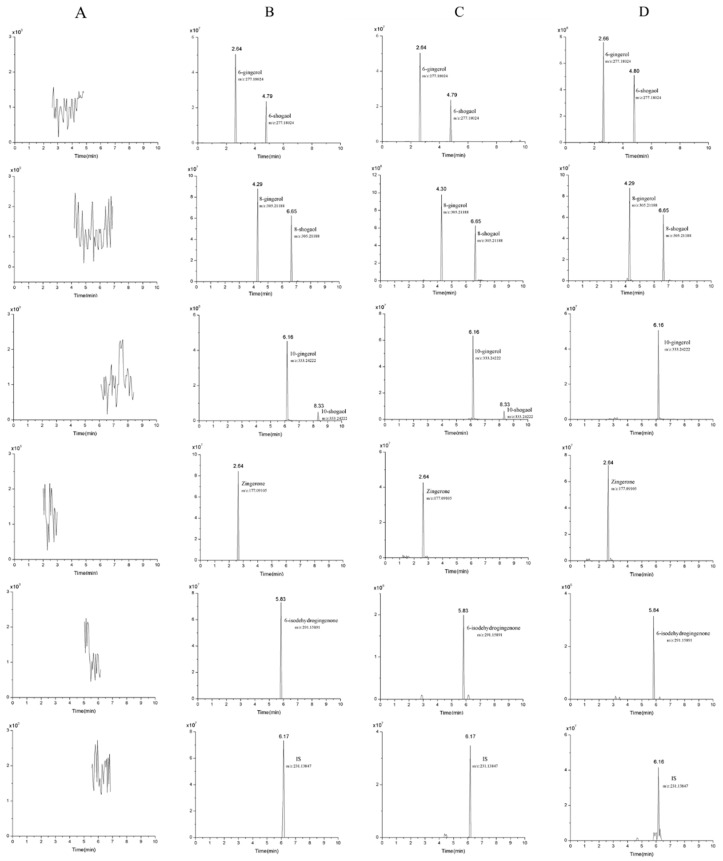
Representative chromatograms of eight analytes and IS: Blank plasma (**A**); blank plasma spiked with analytes and IS (**B**); plasma (**C**) and lung sample (**D**) collected at 1 h after oral administration of ginger extract at a dose of 400 mg/kg.

The selectivity was evaluated by comparing the typical chromatograms of blank plasma, heart, liver, spleen, lung, kidney, stomach, intestine, and brain; blank plasma or organ spiked with the eight target analytes and IS; and bio-samples (plasma and lung tissue) after oral administration of ginger extract. There was no endogenous interference in the full scan mode for each of the analytes in all samples and the representative spectra is presented in Figure 2.

The linearity of the eight target analytes was estimated under optimized conditions. Linear ranges, correlation coefficients (r) and LLOQ are shown in Table S1. The standard curves of the eight target analytes had good linearity with correlation coefficients more than 0.9910. The lowest LLOQ was 1.0 ng/mL.

#### 2.2.2. Precision, Accuracy, Extraction Recovery and Matrix Effect

Intra- and inter-day precision and accuracy of the eight analytes in QC samples are summarized in Table S2. The results indicated that the intra- and inter-day precision (RSD%) of these analytes were in the range of 1.1% to 12.2%, respectively, while the corresponding REs ranged from –8.7% to 8.7%, respectively. These results suggested that this method had an acceptable precision and accuracy [30].

The results for extraction recovery and matrix effect of the eight analytes are also shown in Table S2. The extraction recoveries were in the acceptable range from 91.4% to 107.4%. Regarding the matrix effects, all ratios were between 86.3% and 113.4%, suggesting that there was no endogenous substances and interference for the ionization of the analytes in the co-eluting matrix.

#### 2.2.3. Stability

The stability of the eight analytes in rat plasma and organ tissues under different conditions is shown in Table S3. The extracted samples were stable under the testing conditions including auto-sampler temperature (4 °C) for 24 h, three freeze–thaw cycles and long-term cold storage (–20 °C for 30 days) with RE% values of –9.7–11.3%. The data indicated that the eight ingredients were stable in biological samples stored under various conditions.

### 2.3. Pharmacokinetic Study

**Figure 3:**
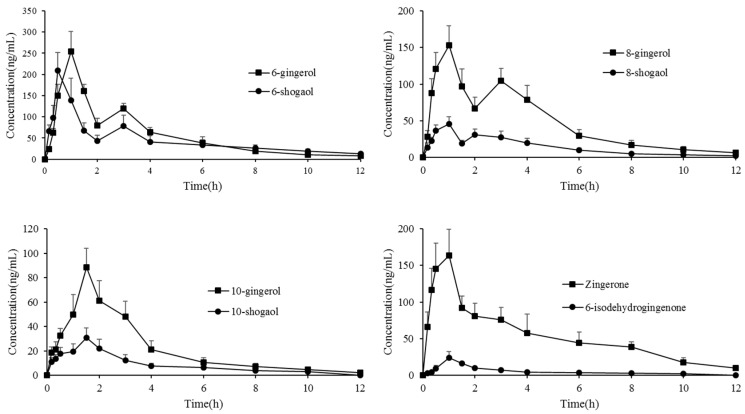
Mean plasma concentration–time curves of eight analytes after oral administration of ginger extract at a dose of 400 mg/kg. (*n* = 10, mean ± SD).

The validated method was successfully utilized to examine the concentrations of 6-gingerol, 6-shogaol, 8-gingerol, 8-shogaol, 10-gingerol, 10-shogaol, Zingerone and 6-isodehydrogingenone in rat plasma and organ samples after oral administration of ginger extract at a dose of 400mg/kg (equivalent to 42.7 mg/kg 6-gingerol, 17.2 mg/kg 6-shogaol, 21.3 mg/kg 8-gingerol, 11.0 mg/kg 8-shogaol, 14.9 mg/kg 10-gingerol, 9.4 mg/kg 10-shogaol, 10.9 mg/kg Zingerone, and 5.8 mg/kg 6-isodehydrogingenone). The mean plasma concentration–time profiles of the eight analytes are presented in Figure 3. The main pharmacokinetic parameters, including the area under the plasma concentration–time curve (AUC_(0–t)_), half-life (t_1/2_), peak time (T_max_), and maximum plasma concentration (C_max_), were estimated with DAS 3.2.8 software using non-compartmental calculations. The results are summarized in Table 1.

Gingerols are aromatic polyphenols, composed of a series of structural analogs, which have an α, β-unsaturated ketone structure with a varied unbranched alkyl chain length [31,32], whereas shogaols are a series of homologues derived from dehydration of gingerol on C-5 and C-4 [33]. Results showed that all analytes were quickly absorbed into the circulatory system with different concentrations; gingerols have similar drug–time curves to shogaols with a corresponding carbon chain length due to structural similarity. The T_max_ of 6-, 8-, and 10-gingerols/shogaols showed that shogaols were absorbed faster than gingerols with a corresponding carbon chain length, although their contents were lower in fresh ginger. Additionally, the increase in the alkyl side chain length weakened the entry capacity in blood of gingerols and shogaols. The t_1/2_ of shogaols were longer than gingerols indicating that shogaols had a relatively long residence time in the body and it may be the reason that shogaols exhibited higher biological activities than gingerols to a certain extent [18,20]. Abnormally, 6-gingerol is the most abundant in ginger, but the detected plasma concentration was not very high (the C_max_ of 6-gingerol was 255.4 ± 44.7 μg/L), probably due to the major existing form of 6-gingerol in plasma is its glucuronide metabolites [26,27], which is consistent with the previous study [27].

Furthermore, 6- and 8-gingerols/shogaols had the double absorption at about 3 h in the concentration–time profile after administration. One reason for the observed result probably resulted from enterohepatic circulation [34] and the other reason might have been the reciprocal transformation between gingerols/shogaols and glucuronide metabolites [26,27]. Additionally, the double-site absorption of drugs might be the first peak expressed by the absorption of the components in the stomach and the second peak was caused by intestinal absorption [35].

The T_max_ of Zingerone was 0.7 ± 0.3 h, showing that Zingerone was quickly absorbed and maintained durably and eliminated slowly in vivo. Thus, further research on Zingerone is under way and needs more attention [36,37]. The T_max_ and C_max_ of 6-isodehydrogingenone were 1.1 ± 0.2 h and 24.4 ± 8.1 μg/L, indicating that although 6-isodehydrogingenone had more active groups in structure, it was still relatively slow to demonstrate its performance in the circulatory system. Its difficulty to enter into the plasma and tissues was possibly due to the decreased abundance of 6-isodehydrogingenone in the ginger extract; moreover, it may also have been related to the binding reaction of other substances.

### 2.4. Tissue Distribution Study

**Figure 4:**
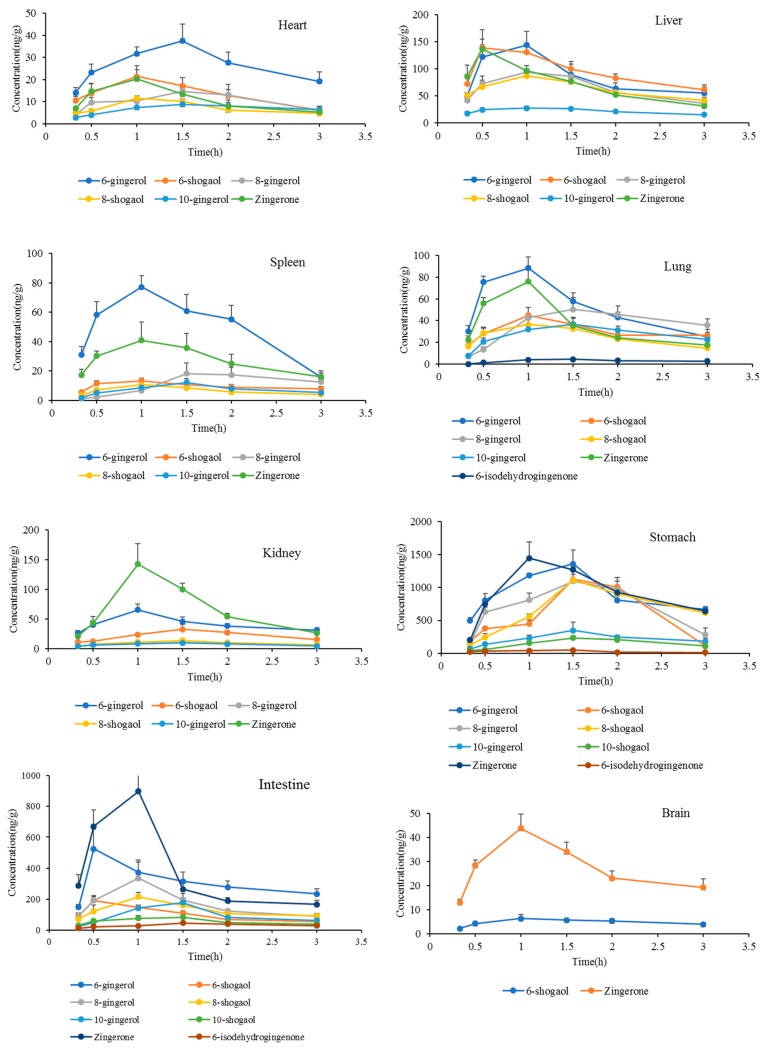
The concentrations of analytes in various tissues at different time points (*n* = 6, mean ± SD).

The tissue distributions of 6-gingerol, 6-shogaol, 8-gingerol 8-shogaol, 10-gingerol, 10-shogaol, Zingerone, and 6-isodehydrogingenone were observed in rats at 0.33, 0.5, 1.0, 1.5, 2, and 3 h after oral administration of ginger extract at a dose of 400 mg/kg (equivalent to 42.7 mg/kg 6-gingerol, 17.2 mg/kg 6-shogaol, 21.3 mg/kg 8-gingerol, 11.0 mg/kg 8-shogaol, 14.9 mg/kg 10-gingerol, 9.4 mg/kg 10-shogaol, 10.9 mg/kg Zingerone, and 5.8 mg/kg 6-isodehydrogingenone). The time point of organ removal referred to the above results obtained from pharmacokinetics. The tissue distribution was determined in various tissues of rats, including the liver, heart, spleen, lung, kidney, stomach, intestine, and brain. The concentrations of ingredients in various tissues at different time points are shown in Figure 4.

The results stated clearly that most of the ingredients could be detected in the studied organizations except for 10-shogaol and 6-isodehydrogingenone, which was probably due to their difficulties to enter into the tissue. In addition, they might have been absorbed slowly and in the time before tissue removal were unable to reach the detectable concentration. Moreover, the hydroxyl and unsaturated double bond groups in 10-shogaol and 6-isodehydrogingenone resulted in a binding reaction with proteins. Interestingly, a trace of 6-isodehydrogingenone was detected in the lung tissue suggesting that it may have potential in the treatment of pulmonary diseases. More research of the reciprocity of 6-isodehydrogingenone and pulmonary diseases would be necessary to prove this.

At 0.33 h after oral administration, different concentrations of 6-gingerol, 6-shogaol, 8-gingerol 8-shogaol, 10-gingerol, and Zingerone were detected in rat tissues, indicating that these ingredients were distributed widely and rapidly in various tissues. With the time extension, concentrations in most of the tissues were obviously decreased at 3 h, indicating that they did not accumulate during the detection period, which was in coincidence with that of the plasma concentration change. 6-gingerol, 6-shogaol, 8-gingerol, 8-shogaol, 10-gingerol and Zingerone were widely distributed in various tissues, especially in the stomach, intestine, liver, lung and kidney, indicating that the digestive system and lung might have been the target organs, and the liver and kidney were the main metabolized and eliminated channel. That is probably the pharmacokinetics basis of 6-gingerol, 6-shogaol, 8-gingerol 8-shogaol, 10-gingerol, and Zingerone for good therapeutic effect on the digestive and respiratory system. 6-shogaol and Zingerone were also detected in the brain suggesting that ginger has potential to treat brain-related diseases due to their penetrability to the blood–brain barrier.

Our ultimate goal is not only to explore the absorption and distribution of gingerols and shogaols in normal organisms, but also to compare normal animals with disease models. This research is now being carried out, and expected to discover new therapeutic targets of ginger for the treatment of diseases.

## 3. Materials and Methods

### 3.1. Materials and Reagents

6-gingerol, 6-shogaol, and 8-gingerol were received from the Henan Province Chinese Medicine Research Institute. 8-shogaol, 10-gingerol, 10-shogaol, Zingerone, and 6-isodehydrogingenone were purchased from Sichuan Vicket Biological Technology Co., Ltd. (Sichuan, China). Atractylenolide I was purchased from the China Research Institute of Food and Drug Verification (Beijing, China). The purity of each reference standard was above 98%. Heparin sodium and sodium carboxymethylcellulose (CMC–Na) were purchased from Sigma–Aldrich (St. Louis, MO, USA). Acetonitrile and methanol of HPLC grade were obtained from Thermo Fisher Scientific (Fair Lawn, NJ, USA). Chromatography grade formic acid was supplied by Sigma–Aldrich (St. Louis, MO, USA). Ethyl acetate was purchased from Tianjin Dasen Chemical Products Sales Co. Ltd. (Tianjin, China). Water used throughout the study was obtained from a MilliQ Reagent Water System (Billerica, MA, USA). Unless otherwise stated, all other chemicals were of analytical grade.

### 3.2. Preparation of Ginger Extract

Ginger was purchased from a farm product market (Zhengzhou, China) and identified as the rhizome of *Z. officinale* by Professor Suiqing Chen at the Henan University of Chinese Medicine. A total of 10 kg of ginger was cut into fine filaments and refluxed with 75% ethanol (1:10, *w*/*v*) twice for 1.5 h. The combined solutions were filtered and concentrated to 0.5 g/mL, and the concentrates were purified by D101 macroporous adsorption resin column chromatography using a gradient elution of 30%, 50%, 70%, and 95% ethanol. The 70% and 95% ethanol eluate were collected and evaporated under vacuum to obtain a gingerol-enriched extract. LC–MS was employed to determine the contents of 6-gingerol, 6-shogaol, 8-gingerol, 8-shogaol, 10-gingerol, 10-shogaol, Zingerone, and 6-isodehydrogingenone in the gingerol-enriched extract; the concentrations of each individual were 106.8, 42.9, 53.2, 27.4, 37.4, 23.5, 27.3, and 14.4 mg/g, respectively.

### 3.3. Animals and Pharmacokinetic Study

#### 3.3.1. Animals

All protocols of animal experiments were performed in accordance with the guidelines of the Committee on the Care and Use of Laboratory Animals in China. Forty-six Wistar rats (weight 200 ± 20 g) were obtained from Beijing Vital River Laboratory Animal Technology Co., Ltd. (Beijing, China, Certificate No.: SCXK (Jing) 2016-0011). The rats were acclimated to standard housing (Henan University of Chinese Medicine, Certificate No.: SCXK (Yu) 2015-0005) and environmental conditions (25 °C, 60% relative humidity) for 1 week. Before administration, rats were fasted for 12 h with access to water freely.

#### 3.3.2. Oral Administration of Ginger Extract

The rats were orally administrated with a single dose of 400 mg/kg ginger extract. The oral dose was calculated on the basis of the human dose in the Chinese Pharmacopoeia (2015) and a pre-experiment which contained a certain amount of target components and could be detected in plasma. Ten rats were used for blood collection, and the other thirty-six rats were used for the collection of tissues. Extract of ginger was suspended in CMC–Na to obtain a stable solution. After administration, blood samples were collected from the orbital venous plexus and placed into heparinized haemospasia tubes at 0, 0.17, 0.33, 0.5, 1, 1.5, 2, 3, 4, 6, 8, 10, and 12 h after oral administration. The blood samples were immediately centrifuged at 4000 rpm for 20 min at 4 °C. The plasma was removed and frozen at −80 °C until analysis. For organ samples, after administration at 0.33, 0.5, 1, 1.5, 2, and 3 h (six rats at each time), the rats were intraperitoneally anesthetized with 10% chloral hydrate and the liver, heart, spleen, lung, kidney, stomach, intestine, and brain were isolated immediately. These collected tissue samples were stored at −80 °C until analysis.

### 3.4. Instruments and Experimental Conditions

Chromatographic analysis was performed on a Thermo UltiMate 3000 UPLC system with a conditioned auto-sampler equipped with Syncronis C18 column (100 × 2.1 mm, 1.7 µm, Thermo Fisher Scientific) and the column temperature was maintained at 30 °C. A gradient elution with water containing 0.1% formic acid (A) and acetonitrile (B) was used at a flow rate of 0.25 mL/min as follows: 0–6 min (60%–95% B); 6–9 min (95% B); 9.1–12 min (60% B). The injection volume was 5 μL.

Mass spectrometric detection was performed using a Thermo Q-Exactive-Qibitrap-MS (Thermo Scientific, Waltham, MA, USA) with an electrospray Ionization (ESI) interface in the positive ionization mode at full scan mode. The scope of data acquisition is *m*/*z* 100.0–1000.0. The optimal MS parameters were as follows: Spray voltage 3.8 Kv (+); sheath gas flow rate, 40 arbitrary units; auxiliary gas flow rate, 10 arbitrary units; capillary temperature and auxiliary gas heater temperature, 350 °C. The collision gas (N_2_) for MS was maintained at 10 mL/min.

### 3.5. Sample Preparation

#### 3.5.1. Calibration Standards and Quality Control Samples

The mixture stock solution of 6-gingerol, 6-shogaol, 8-gingerol, 8-shogaol, 10-gingerol, 10-shogaol, Zingerone, and 6-isodehydrogingenone were prepared by dissolving the proper amounts of each standard substance in 10 mL of methanol to yield concentrations of 416.7, 484.0, 1104.9, 471.6, 412.4, 400.0, 749.1, and 322.1 μg/mL, respectively. Stock solutions were prepared and serially diluted to give working solutions used for validation experiments. The internal standard (IS) stock solution (667 g/mL) was prepared by dissolving the proper amount of Atractylenolide I in methanol. A 100 ng/mL working standard solution of IS was prepared by dilution with methanol. Calibration standard samples were prepared by spiking blank plasma and tissues homogenate with appropriate amounts of working solutions, yielding concentrations of 2.0–500.0 ng/mL for 6-gingerol, 6-shogaol, 8-gingerol, 8-shogaol, 10-gingerol and Zingerone, and 1.0–250.0 ng/mL for 10-shogaol and 6-isodehydrogingenone. Quality control (QC) samples were independently prepared in the same way at 62.5, 250.0, and 500.0 ng/mL for 6-gingerol, 6-shogaol, 8-gingerol, 10-gingerol, and Zingerone; 15.6, 125.0, and 250.0 ng/mL for 8-shogaol, 10-shogaol and 6-isodehydrogingenone. The standard and quality control samples were extracted on each analysis day using the same procedures for plasma samples as described above.

#### 3.5.2. Blood Samples

The plasma (200 μL) was spiked with 20 μL of IS (100 ng/mL) and 600 μL methanol vortexed for 1 min to precipitate proteins. The mixture was extracted by adding 800 μL ethyl acetate and oscillating at room temperature for 5 min. After centrifugation for 10 min at 12,000 rpm, the supernatant was transferred and evaporated to dry under a mild nitrogen flow at 40 °C. The residue was dissolved in 100 μL methanol and centrifuged at 12,000 rpm for 10 min. Then 80 μL of supernatant were transferred to sampling vials for the LC–MS system.

#### 3.5.3. Tissue Samples

The thawed tissue samples (1.0 g) were homogenized in normal saline (2 mL), and diluted of homogenate with 6 mL methanol, and then mixed with 20 μL of IS solution (100 ng/mL). A total of 5 mL supernatant was removed and extracted by adding 5 mL ethyl acetate and vortexed for 5 min, then centrifuged at 12,000 rpm for 10 min. The above operation was repeated, and then the two supernatants were combined and blow dried with nitrogen at 40 °C. The residue was dissolved in 300 μL methanol and centrifuged at 12,000 rpm for 10 min. The supernatant was filtered through a 0.22 μm filter. A total of 5 μL of filtrate was subjected to the LC–MS system for analysis.

### 3.6. Method Validation

According to FDA guidance for validation of bioanalytical methods, the UPLC–Q-Exactive–HRMS was established and validated for selectivity, linearity, precision, accuracy, matrix effect, extraction recovery, and stability of the eight analytes in rat plasma and tissues [30].

## 4. Conclusions

A sensitive, rapid, and reliable UPLC–Q-Exactive–HRMS analytical method was established and validated for the simultaneous determination and quantification of 6-gingerol, 6-shogaol, 8-gingerol, 8-shogaol, 10-gingerol, 10-shogaol, Zingerone, and 6-isodehydrogingenone in the plasma and tissues of rats. The results demonstrated that of the active ingredients 6-gingerol, 6-shogaol, 8-gingerol, 8-shogaol, 10-gingerol, and Zingerone were absorbed rapidly into the circulatory system, while the other components such as 10-shogaol and 6-isodehydrogingenone had relatively difficulty being absorbed after oral administration of ginger extract. In addition, 6-shogaol and Zingerone were able to penetrate the blood–brain barrier and enter the brain. These results demonstrated the pharmacokinetics and tissue distribution of active ingredients of ginger in vivo, and provided useful information for further pharmacological and clinical practice use of ginger.

## Figures and Tables

**Table 1:** Pharmacokinetic parameters of eight constituents in rats plasma after oral administration of ginger extract at a dose of 400 mg/kg (*n* = 10, mean ± SD).

Analytes	AUC_(0–t)_ (μg/L·h)	t_1/2_ (h)	T_max_ (h)	C_max_ (μg/L)
6-gingerol	691.5 ± 80.7	2.6 ± 0.7	0.9 ± 0.2	255.4 ± 44.7
6-shogaol	545.3 ± 95.7	3.9 ± 1.2	0.7 ± 0.3	214.4 ± 40.7
8-gingerol	579.4 ± 79.4	2.7 ± 0.3	1.1 ± 0.2	156.0 ± 23.5
8-shogaol	171.3 ± 25.9	3.0 ± 1.2	0.9 ± 0.2	46.5 ± 9.7
10-gingerol	259.1 ± 46.0	2.7 ± 0.4	1.5 ± 0.3	92.8 ± 11.3
10-shogaol	101.3 ± 24.6	2.5 ± 2.4	1.4 ± 0.4	32.5 ± 6.2
Zingerone	641.0 ± 85.3	2.2 ± 0.4	0.7 ± 0.3	174.8 ± 23.0
6-isodehydrogingenone	63.7 ± 9.7	1.0 ± 1.1	1.1 ± 0.2	24.4 ± 8.1

## Supplementary Materials

The Supplementary Materials are available online.
