# Supplementary material for: Pharmacokinetics and Tissue Distribution of Gingerols and Shogaols from Ginger (Zingiber officinale Rosc.) in Rats by UPLC–Q-Exactive–HRMS
Source: Molecules. 2019 Jan 31;24(3):512. doi: 10.3390/molecules24030512 (PMC6384666; doi:10.3390/molecules24030512)
Supplement: Supplementary file 1 [file molecules-24-00512-s001.pdf]

Supplementary Material

Pharmacokinetics and tissue distribution of gingerols and shogaols from ginger *(Zingiber officinale* Rosc.) in rats by UPLC-Q-Exactive-HRMS

Ling-ling Li[1,2], Ying Cui[1,2*], Xing-han Guo[1], Kai Ma[3], Ping Tian[3], Jing Feng[1], and Jun-Ming Wang[1]

[1] School of Pharmacy, Henan University of Chinese Medicine, 156 Jinshui east Road, Zhengzhou 450046, China; openleeling@163.com (L.L.); gxh5508@163.com (X.G.); fj.xyz@163.com (J.F.); mjw98@126.com (J.W.)

[2] Collaborative Innovation Center for Respiratory Disease Diagnosis and Treatment & Chinese Medicine Development of Henan Province, 156 Jinshui east Road, Zhengzhou 450046, China

[3] Henan Province Chinese Medicine Research Institute; zzmk1968@126.com (K.M.); tianping082@163.com (P.T.)

[*] Correspondence: cyexin@hactcm.edu.cn (Y.C.); Tel.: +86-371-8653-5313 (Y.C.)

Table S1: Summary of regression equations, linear ranges, correlation coefficients and LLOQ of eight analytes in rat plasma and tissues.

Analytes	Plasma/tissues	Regression equation	Linear range (ng/mL)	r	LLOQ (ng/mL)
6-gingerol	Plasma	$Y=4.4\times10^{-3}X+2.4\times10^{-3}$	2.0-500.0	0.9992	2.0
	Heart	$Y=6.1\times10^{-4}X+1.9\times10^{-3}$	2.0-500.0	0.9963	2.0
	Liver	$Y=3.8\times10^{-4}X+4.8\times10^{-3}$	2.0-500.0	0.9982	2.0
	Spleen	$Y=7.7\times10^{-4}X+5.1\times10^{-3}$	2.0-500.0	0.9995	2.0
	Lung	$Y=5.8\times10^{-4}X+2.4\times10^{-3}$	2.0-500.0	0.9946	2.0
	Kidney	$Y=9.9\times10^{-4}X+2.2\times10^{-3}$	2.0-500.0	0.9976	2.0
	Stomach	$Y=5.3\times10^{-4}X+9.2\times10^{-3}$	39.9-5000.0	0.9995	39.9
	Intestine	$Y=8.3\times10^{-4}X+1.0\times10^{-2}$	15.6-2000.0	0.9970	15.6
	Brain	$Y=5.0\times10^{-4}X+1.1\times10^{-2}$	2.0-500.0	0.9976	2.0
6-shogaol	Plasma	$Y=1.9\times10^{-3}X+1.4\times10^{-3}$	2.0-500.0	0.9988	2.0
	Heart	$Y=2.7\times10^{-3}X+1.3\times10^{-3}$	2.0-500.0	0.9964	2.0
	Liver	$Y=1.9\times10^{-3}X+1.9\times10^{-3}$	2.0-500.0	0.9957	2.0
	Spleen	$Y=3.3\times10^{-3}X+1.5\times10^{-3}$	2.0-500.0	0.9948	2.0
	Lung	$Y=1.9\times10^{-3}X+5.5\times10^{-3}$	2.0-500.0	0.9961	2.0
	Kidney	$Y=4.3\times10^{-3}X+6.5\times10^{-3}$	2.0-500.0	0.9987	2.0
	Stomach	$Y=2.3\times10^{-3}X+4.5\times10^{-3}$	39.9-5000.0	0.9991	39.9
	Intestine	$Y=4.8\times10^{-3}X+2.4\times10^{-3}$	15.6-2000.0	0.9986	15.6
	Brain	$Y=1.8\times10^{-3}X+2.9\times10^{-3}$	2.0-500.0	0.9954	2.0
8-gingerol	Plasma	$Y=1.2\times10^{-3}X +2.8\times10^{-4}$	2.0-500.0	0.9946	2.0
	Heart	$Y=3.0\times10^{-4}X +2.9\times10^{-3}$	2.0-500.0	0.9910	2.0
	Liver	$Y=4.9\times10^{-4}X -3.2\times10^{-3}$	2.0-500.0	0.9949	2.0
	Spleen	$Y= 3.0\times10^{-4}X -2.8\times10^{-3}$	2.0-500.0	0.9941	2.0
	Lung	$Y=4.9\times10^{-4}X -5.7\times10^{-3}$	2.0-500.0	0.9943	2.0
	Kidney	$Y=2.3\times10^{-4}X -5.6\times10^{-4}$	2.0-500.0	0.9960	2.0

	Stomach	$Y=8.2\times10^{-4}X -2.1\times10^{-3}$	39.9-5000.0	0.9941	39.9
	Intestine	$Y=4.6\times10^{-4}X +1.4\times10^{-3}$	15.6-2000.0	0.9952	15.6
	Brain	$Y=5.8\times10^{-4}X +2.6\times10^{-3}$	2.0-500.0	0.9960	2.0
8-shogaol	Plasma	$Y=6.2\times10^{-4}X+1.1\times10^{-3}$	2.0-500.0	0.9941	2.0
	Heart	$Y=4.4\times10^{-4}X+2.6\times10^{-3}$	2.0-500.0	0.9986	2.0
	Liver	$Y=4.2\times10^{-4}X+5.1\times10^{-3}$	2.0-500.0	0.9925	2.0
	Spleen	$Y=6.7\times10^{-4}X-1.9\times10^{-3}$	2.0-500.0	0.9938	2.0
	Lung	$Y=5.8\times10^{-4}X+3.7\times10^{-3}$	2.0-500.0	0.9994	2.0
	Kidney	$Y=3.6\times10^{-4}X+2.1\times10^{-3}$	2.0-500.0	0.9968	2.0
	Stomach	$Y=3.9\times10^{-4}X+2.6\times10^{-3}$	39.9-5000.0	0.9956	39.9
	Intestine	$Y=5.6\times10^{-4}X-1.7\times10^{-3}$	15.6-2000.0	0.9984	15.6
	Brain	$Y=4.7\times10^{-4}X-2.2\times10^{-3}$	2.0-500.0	0.9928	2.0
10-gingerol	Plasma	$Y=1.3\times10^{-3}X+ 3.7\times10^{-3}$	2.0-500.0	0.9996	2.0
	Heart	$Y=4.0\times10^{-4}X-1.5\times10^{-4}$	2.0-500.0	0.9910	2.0
	Liver	$Y=3.7\times10^{-4}X+6.1\times10^{-4}$	2.0-500.0	0.9937	2.0
	Spleen	$Y=5.6\times10^{-4}X-2.5\times10^{-4}$	2.0-500.0	0.9942	2.0
	Lung	$Y=4.4\times10^{-4}X+6.8\times10^{-4}$	2.0-500.0	0.9963	2.0
	Kidney	$Y=4.0\times10^{-4}X-1.5\times10^{-4}$	2.0-500.0	0.9969	2.0
	Stomach	$Y=3.0\times10^{-4}X+4.8\times10^{-3}$	39.9-5000.0	0.9942	39.9
	Intestine	$Y=3.9\times10^{-4}X+3.2\times10^{-3}$	15.6-2000.0	0.9943	15.6
	Brain	$Y=2.9\times10^{-4}X+6.5\times10^{-3}$	2.0-500.0	0.9950	2.0
10-shogaol	Plasma	$Y=1.8\times10^{-6}X-2.3\times10^{-6}$	1.0-250.0	0.9985	1.0
	Heart	$Y=2.3\times10^{-6}X+4.3\times10^{-5}$	1.0-250.0	0.9965	1.0
	Liver	$Y=2.8\times10^{-6}X-2.0\times10^{-5}$	1.0-250.0	0.9937	1.0
	Spleen	$Y=2.4\times10^{-6}X+1.7\times10^{-5}$	1.0-250.0	0.9991	1.0
	Lung	$Y=1.6\times10^{-6}X+3.7\times10^{-5}$	1.0-250.0	0.9963	1.0

	Kidney	$Y=4.4\times10^{-6}X+6.7\times10^{-5}$	1.0-250.0	0.9948	1.0
	Stomach	$Y=2.8\times10^{-6}X+2.1\times10^{-5}$	15.6-2000.0	0.9918	15.6
	Intestine	$Y=2.9\times10^{-6}X+2.7\times10^{-5}$	15.6-2000.0	0.9941	15.6
	Brain	$Y=4.9\times10^{-6}X-1.9\times10^{-5}$	1.0-250.0	0.9966	1.0
Zingerone	Plasma	$Y=6.5\times10^{-3}X+3.3\times10^{-2}$	2.0-500.0	0.9977	2.0
	Heart	$Y=5.5\times10^{-4}X+5.7\times10^{-3}$	2.0-500.0	0.9984	2.0
	Liver	$Y=3.4\times10^{-4}X-2.7\times10^{-3}$	2.0-500.0	0.9990	2.0
	Spleen	$Y=4.4\times10^{-4}X+5.6\times10^{-3}$	2.0-500.0	0.9994	2.0
	Lung	$Y=5.1\times10^{-4}X-4.2\times10^{-3}$	2.0-500.0	0.9934	2.0
	Kidney	$Y =4.9\times10^{-4}X+4.4\times10^{-3}$	2.0-500.0	0.9954	2.0
	Stomach	$Y= 4.0\times10^{-4}X+8.5\times10^{-3}$	39.9-5000.0	0.9992	39.9
	Intestine	$Y=4.5\times10^{-4}X-1.0\times10^{-2}$	15.6-2000.0	0.9953	15.6
	Brain	$Y=2.9\times10^{-4}X+8.4\times10^{-3}$	2.0-500.0	0.9970	2.0
6-isodehydrogingenone	Plasma	$Y=1.3\times10^{-2}X-1.2\times10^{-2}$	1.0-250.0	0.9988	1.0
	Heart	$Y=4.8\times10^{-2}X+1.7\times10^{-2}$	1.0-250.0	0.9994	1.0
	Liver	$Y=2.3\times10^{-2}X-4.2\times10^{-3}$	1.0-250.0	0.9944	1.0
	Spleen	$Y=3.8\times10^{-2}X+1.9\times10^{-2}$	1.0-250.0	0.9931	1.0
	Lung	$Y=5.8\times10^{-2}X+1.2\times10^{-2}$	1.0-250.0	0.9954	1.0
	Kidney	$Y=3.4\times10^{-2}X+6.8\times10^{-3}$	1.0-250.0	0.9941	1.0
	Stomach	$Y=6.5\times10^{-3}X-1.4\times10^{-3}$	7.8-2000.0	0.9985	7.8
	Intestine	$Y=8.9\times10^{-2}X+3.1\times10^{-2}$	7.8-2000.0	0.9958	7.8
	Brain	$Y=4.8\times10^{-3}X+2.2\times10^{-2}$	1.0-250.0	0.9927	1.0

X represented the concentration (ng/mL) and Y represented the area ratio.

Table S2: The intra-day and inter-day precisions and accuracies, extraction recoveries, matrix effects of the eight analytes in rat plasma and tissues (n = 6).

Analytes	Plasma/ tissues	C_{nom} (ng/mL)	Intra-day			Inter-day			Extraction recovery Mean ± S.D. (%)	Matrix effect Mean ± S.D. (%)
			C_{obs} (ng/mL)	Precision (RSD%)	Accuracy (RE%)	C_{obs} (ng/mL)	Precision (RSD%)	Accuracy (RE%)		
6-gingerol	Plasma	62.5	66.8	4.8	6.9	68.0	5.3	8.7	103.8±3.9	95.3±5.5
		250.0	242.8	2.6	-2.9	245.1	2.5	-2.0	94.6±4.3	97.2±2.3
		500.0	485.4	3.8	-2.9	484.7	3.7	-3.1	96.9±5.4	100.5±4.6
	Heart	62.5	66.4	3.7	6.2	67.6	4.9	8.2	107.4±3.9	97.2±5.6
		250.0	246.4	2.9	-1.5	245.8	2.6	-1.7	97.2±2.0	96.2±2.1
		500.0	488.7	5.8	-2.3	487.0	4.6	-2.6	97.0±4.2	97.7±5.1
	Liver	62.5	66.2	3.4	5.9	66.9	4.9	7.0	97.3±3.4	98.1±5.7
		250.0	243.5	3.1	-2.6	245.3	2.8	-1.9	97.4±2.0	96.6±2.6
		500.0	488.7	4.8	-2.3	486.9	3.9	-2.6	97.3±3.4	100.9±5.0
	Spleen	62.5	66.2	3.4	5.9	67.2	4.0	7.5	107. 8±4.0	98.5±4.0
		250.0	244.3	3.6	-2.3	245.1	2.5	-2.0	98.3±2.0	95.6±1.5
		500.0	491.3	3.7	-1.7	488.4	4.1	-2.3	97.1±4.1	100.4±4.7
	Lung	62.5	65.5	6.2	4.8	67.1	6.1	7.3	107.0±5.8	101.1±4.8
		250.0	243.8	3.3	-2.5	245.8	2.9	-1.7	98.0±2.5	93.9±5.3
		500.0	486.7	4.2	-2.7	486.3	4.2	-2.8	98.3±3.7	95.9±2.2
	Kidney	62.5	66.2	3.4	5.9	67.5	4.7	8.0	107.5±4.3	101.0±4.5
		250.0	243.8	3.3	-2.5	245.8	2.6	-1.7	98.0±2.5	99.5±5.4
		500.0	486.6	4.1	-2.7	485.8	4.0	-2.8	96.3±3.5	97.1±2.7
	Stomach	62.5	66.5	5.5	6.4	67.8	5.8	8.4	109.8±6.2	100.2±4.4
		250.0	243.2	3.6	-2.7	245.5	2.9	-1.8	99.3±2.6	96.1±6.8
		500.0	482.4	4.7	-3.5	482.9	4.1	-3.4	97.1±3.5	97.0±3.2
	Intestine	62.5	66.2	3.3	5.9	67.7	5.1	8.2	109.2±4.7	100.8±4.0

		250.0	244.5	3.7	-2.2	245.2	2.9	-1.9	98.2±2.7	99.9±5.7
		500.0	484.7	4.1	-3.1	485.0	4.0	-3.0	96.8±3.6	96.2±2.7
	Brain	62.5	66.4	8.4	6.2	67.9	6.6	8.6	109.8±6.1	98.3±3.6
		250.0	242.9	3.7	-2.8	245.0	3.0	-2.0	98.4±2.6	96.3±5.0
		500.0	481.3	5.0	-3.7	482.9	4.2	-3.4	96.7±3.7	97.2±2.9
6-shogaol	Plasma	62.5	63.7	2.1	1.9	63.9	2.3	2.2	102.3±1.7	98.0±1.5
		250.0	245.1	3.5	-2.0	247.1	5.1	-1.2	97.8±4.0	96.3±4.7
		500.0	481.7	4.0	-3.7	486.1	4.2	-2.8	94.1±4.1	99.4±4.9
	Heart	62.5	63.0	2.1	0.8	63.4	2.7	1.5	101.5±2.6	98.2±2.8
		250.0	243.7	3.2	-2.5	246.3	4.9	-1.5	98.9±5.1	96.5±4.5
		500.0	490.2	3.7	-2.0	488.2	4.3	-2.4	97.2±3.7	96.9±4.0
	Liver	62.5	63.4	4.4	1.4	64.0	3.4	2.4	101.1±2.9	96.5±3.3
		250.0	245.4	3.0	-1.8	248.3	4.7	-0.7	97.9±4.0	97.3±4.7
		500.0	488.3	3.8	-2.3	487.3	4.0	-2.5	96.9±4.1	100.1±4.7
	Spleen	62.5	64.0	2.9	2.4	64.0	2.5	2.4	102.5±2.5	98.2±5.4
		250.0	246.7	3.5	-1.3	246.6	4.4	-1.4	97.9±3.6	96.0±2.2
		500.0	482.7	4.3	-3.5	485.3	4.6	-2.9	97.0±4.5	95.1±3.5
	Lung	62.5	64.0	2.1	2.4	63.9	2.1	2.2	101.8±2.2	99.3±4.3
		250.0	244.4	3.9	-2.2	246.5	5.4	-1.4	97.7±4.8	97.6±2.3
		500.0	483.3	4.2	-3.3	486.3	4.2	-2.7	97.6±3.5	95.5±4.8
	Kidney	62.5	63.4	2.7	1.4	63.6	2.8	1.7	102.3±2.0	100.4±4.6
		250.0	245.7	3.9	-1.7	247.1	5.3	-1.2	99.3±4.3	96.9±3.1
		500.0	476.5	5.0	-4.7	484.2	4.5	-3.2	95.9±4.2	96.4±6.0
	Stomach	62.5	63.3	3.1	1.2	63.7	2.8	1.9	103.4±1.4	101.5±3.5
		250.0	244.1	4.4	-2.4	246.5	5.4	-1.4	100.6±4.9	98.1±2.8
		500.0	481.1	4.6	-3.8	486.6	4.5	-2.7	98.5±3.9	97.5±5.8

	Intestine	62.5	64.1	3.1	2.6	64.1	2.7	2.6	102.3±3.1	97.4±4.9
		250.0	244.3	4.6	-2.3	246.7	5.2	-1.3	97.7±5.0	96.9±2.2
		500.0	480.6	5.6	-3.9	484.3	4.9	-3.1	95.1±4.5	97.1±5.4
	Brain	62.5	62.6	1.9	0.2	63.3	3.2	1.3	101.9±3.4	97.7±5.1
		250.0	243.6	4.3	-2.5	246.4	5.5	-1.5	99.1±5.6	100.1±4.0
		500.0	477.1	5.7	-4.6	485.6	5.0	-2.9	98.0±4.4	95.2±5.4
8-gingerol	Plasma	62.5	60.9	5.8	-2.5	61.7	4.6	-1.3	96.9±3.5	93.4±4.1
		250.0	255.5	2.6	2.2	256.3	2.3	2.5	101.5±1.8	96.9±2.3
		500.0	478.2	2.2	-4.4	488.4	3.3	-2.3	97.9±3.6	99.6±3.9
	Heart	62.5	60.6	6.3	-3.0	61.5	5.0	-1.7	98.0±5.1	92.9±4.4
		250.0	257.5	3.8	3.0	257.6	3.1	3.0	102.9±2.9	94.9±2.8
		500.0	472.9	3.2	-5.4	487.7	4.2	-2.5	94.1±2.7	98.4±4.5
	Liver	62.5	60.3	5.5	-3.6	61.8	4.6	-1.1	98.3±3.2	95.4±4.5
		250.0	254.8	2.2	1.9	256.4	2.5	2.6	101.9±1.7	97.8±2.8
		500.0	484.9	2.8	-3.0	491.7	3.2	-1.7	98.9±3.0	100.6±3.8
	Spleen	62.5	60.6	6.4	-3.0	61.7	5.1	-1.3	99.2±4.0	98.3±4.1
		250.0	254.5	3.1	1.8	257.1	2.4	2.8	103.3±2.0	93.9±3.8
		500.0	481.6	3.3	-3.7	490.6	3.7	-1.9	99.0±3.5	97.9±2.0
	Lung	62.5	60.6	6.4	-3.0	61.0	5.9	-2.4	96.7±5.7	96.9±3.5
		250.0	256.8	3.4	2.7	257.2	2.9	2.9	102.6±2.5	91.7±4.9
		500.0	481.6	3.3	-3.7	488.4	3.4	-2.3	97.9±3.0	95.2±1.9
	Kidney	62.5	60.3	7.0	-3.6	61.1	5.8	-2.2	98.0±5.2	98.5±3.5
		250.0	254. 6	3.1	1.8	255.6	2.7	2.3	102.4±2.6	93.5±6.3
		500.0	479.5	2.6	-4.1	487.7	3.5	-2. 5	96.9±2.9	97.2±2.9
	Stomach	62.5	60.6	6.4	-3.1	61.4	5.2	-1.8	100.0±4.8	97.7±4.0
		250.0	254.7	3.0	1.9	255.7	2.7	2.3	102.9±2.7	94.3±5.4

		500.0	477.2	3.9	-4. 6	488.2	3.8	-2.4	99.3±2.9	97.0±3.0
	Intestine	62.5	60.1	7.3	-3.8	61.3	5.5	-2.0	98.7±4.5	97.9±4.3
		250.0	255.8	2.8	2.3	256.1	2.6	2.4	102.8±2.5	94.2±5.7
		500.0	473.9	4.4	-5.2	485.9	4.2	-2.8	98.4±3.2	97.3±2.4
	Brain	62.5	60.4	6.8	-3.4	61.3	5.5	-2.0	98.7±4.8	96.3±3.4
		250.0	255.7	3.5	2.3	256.2	2.5	2.5	102.6±2.1	95.4±4.4
		500.0	477.0	5.6	-4.6	486.7	4.4	-2.7	98.3±3.5	96.9±1.9
8-shogaol	Plasma	15.6	15.9	11.4	1.5	15.8	9.5	1.1	102.3±8.2	112.7±9.6
		125.0	118.3	7.8	-5.4	121.3	5.8	-3.0	95.5±4.6	93.3±5.7
		250.0	259.0	5.4	3.6	259.4	4.4	3.7	104.2±4.5	99.4±4.7
	Heart	15.6	15.5	8.3	-0.7	15.8	9.9	1.1	101.4±9.5	111.7±8.3
		125.0	117.9	7.3	-5.7	121.0	5.6	-3.2	95.9±5.2	93.6±5.9
		250.0	257.4	4.7	2.9	258.6	4.2	3.4	103.5±4.0	98.9±4.7
	Liver	15.6	16.2	11.3	3.6	16.0	9.2	2.5	105.6±8.2	111.5±8.3
		125.0	121.6	7.5	-2.7	123.5	5.5	-1.2	98.8±3.9	95.9±5.7
		250.0	256.7	4.4	2.7	257.5	3.7	3.0	101.7±3.8	98.3±3.8
	Spleen	15.6	16.5	12.2	5.8	16.1	10.0	3.3	102.0±9.0	112.9±10.0
		125.0	121.6	5.7	-2.7	123.5	4.4	-1.2	99.8±3.8	95.2±4.8
		250.0	256.7	4.4	2.7	257.9	4.5	3.2	103.4±4.7	97.3±5.3
	Lung	15.6	16.2	8.7	3.6	16.0	8.8	2.3	103.1±8.3	105.9±9.3
		125.0	120.6	6.0	-3.5	121.8	4.9	-2.5	96.3±4.3	93.0±3.8
		250.0	258.4	5.3	3.3	258.9	4.3	3.6	103.3±4.4	99.6±4.8
	Kidney	15.6	16.2	14.2	3.4	16.0	10.0	2.3	102.9±9.9	113.6±11.9
		125.0	119.4	7.3	-4.5	121.5	5.7	-2.8	97.9±5.5	93.1±5.8
		250.0	259.4	5.4	3.7	259.4	4.5	3.7	103.9±4.2	98.4±4.9
	Stomach	15.6	15.6	8.7	-0.4	15.6	9.6	0.1	96.6±8.5	107.9±10.1

		125.0	119.1	7.4	-4.8	121.5	6.1	-2.8	100.2±4.2	94.4±6.6
		250.0	258.7	5.6	3.5	258.9	4.7	3.6	105.1±3.1	100.2±4.3
	Intestine	15.6	15.4	9.0	-1.5	15.5	10.0	-0.6	101.1±10.8	110.7±11.1
		125.0	119.5	6.8	-4.4	121.7	5.3	-2.6	97.2±4.7	94.9±5.3
		250.0	258.9	4.8	3.5	259.0	4.1	3.6	102.1±4.0	98.8±4.0
	Brain	15.6	15.5	11.1	-0.6	15.8	10.1	0.8	101.6±9.8	113.4±12.3
		125.0	118.8	8.5	-5.0	121.5	6.3	-2.8	98.3±4.9	94.0±5.6
		250.0	257.3	6.5	2.9	258.5	4.9	3.4	103.7±4.3	96.7±4.9
10-gingerol	Plasma	62.5	65.9	4.0	5.5	66.0	3.4	5.6	104.0±2.9	95.2±3.2
		250.0	252.7	4.1	1.1	253.0	5.1	1.2	95.9±3.4	95.1±4.1
		500.0	484.5	2.9	-3.1	504.8	3.7	1.0	98.7±5.8	101.5±4.0
	Heart	62.5	63.9	3.2	2.3	64.7	3.5	3.5	103.6±3.6	104.9±3.7
		250.0	249.7	4.1	-0.1	251.4	4.8	0.5	100.7±4.5	95.4±5.2
		500.0	481.2	2.6	-3.8	503.3	3.9	0.7	100.3±4.2	101.1±4.2
	Liver	62.5	65.6	3.4	4.9	65.4	2.5	4.6	104.2±1.9	96.1±2.3
		250.0	252.7	4.1	1.1	251.6	4.4	0.6	99.4±4.2	94.5±2.9
		500.0	487.2	3.4	-2.6	504.0	3.4	0.8	102.1±2.1	101.2±3.4
	Spleen	62.5	64.6	2.4	3.3	65.1	2.9	4.2	104.7±3.0	103.1±2.0
		250.0	252.4	2.8	0.9	253.1	5.0	1.3	100.7±4.9	102.1±3.1
		500.0	487.2	3.4	-2.6	504.6	3.5	0.9	101.6±2.0	95.9±4.9
	Lung	62.5	64.6	5.2	3.3	65.2	3.5	4.4	104.5±4.1	99.0±4.4
		250.0	254.0	5.0	1.6	253.7	5.4	1.5	100.5±5.5	94.6±3.5
		500.0	484.5	2.9	-3.1	493.9	4.0	-1.2	97.2±3.6	92.9±4.9
	Kidney	62.5	66.2	4.7	6.0	66.2	3.7	6.0	105.8±3.6	101.7±4.5
		250.0	252.2	4.3	0.9	252.4	5.4	1.0	102.2±3.8	95.6±3.7
		500.0	482.3	2.6	-3.5	503.7	3.8	0.7	99.8±3.8	94.7±6.0

	Stomach	62.5	65.5	3.3	4.9	65.9	3.3	5.4	105.7±3.5	101.5±5.0
		250.0	253.4	4.5	1.4	253.0	5.3	1.2	103.4±4.5	97.7±3.9
		500.0	482.0	4.2	-3.6	503.6	4.2	0.7	102.1±2.5	94.9±5.8
	Intestine	62.5	66.6	4.6	6.6	66.0	3.3	5.6	106.1±3.0	102.1±5.1
		250.0	252.0	4.4	0.8	252.6	5.3	1.0	100.0±5.4	96.4±3.2
		500.0	481.5	4.0	-3.7	502.4	4.4	0.5	101.0±3.1	96.8±4.4
	Brain	62.5	65.6	5.0	4.9	65.6	4.6	4.9	104.9±4.7	103.8±2.6
		250.0	251.6	4.6	0.7	252.4	5.4	1.0	101.1±5.7	97.4±2.6
		500.0	481.1	4.5	-3.8	504.0	4.4	0.8	103.1±2.0	95.8±5.7
10-shogaol	Plasma	15.6	14.9	3.0	-4.9	15.2	6.7	-2.7	93.7±2.9	97.9±6.0
		125.0	118.3	2.0	-5.4	118.8	5.0	-5.0	90.2±4.3	97.9±5.5
		250.0	245.4	1.5	-1.8	247.5	3.9	-1.0	98.4±1.7	99.9±4.1
	Heart	15.6	15.2	3.3	-2.8	15.4	7.8	-1.3	98.5±7.4	102.5±8.6
		125.0	121.6	5.6	-2.7	121.0	5.9	-3.2	96.9±6.0	100.8±7.2
		250.0	245.1	1.7	-2.0	247.0	4.1	-1.2	99.4±4.1	99.0±4.9
	Liver	15.6	14.5	6.2	-7.0	15.2	7.5	-2.7	97.2±6.0	97.9±7.4
		125.0	119.6	3.1	-4.3	120.1	4.2	-3.9	95.4±3.0	99.5±4.8
		250.0	247.1	2.5	-1.2	247.0	3.1	-1.2	97.9±2.4	97.3±2.5
	Spleen	15.6	15.3	6.1	-1.9	15.1	6.0	-3.2	98.5±5.8	92.2±5.5
		125.0	118.6	6.5	-5.1	119.5	5.3	-4.4	96.0±4.6	94.1±4.4
		250.0	243.1	3.1	-2.8	246.5	4.0	-1.4	99.0±4.0	99.3±4.4
	Lung	15.6	15.5	10.7	-0.8	15.5	9.2	-0.7	99.3±7.9	86.3±6.1
		125.0	116.9	3.9	-6. 5	118.1	5.6	-5.5	93.6±4.6	96.6±5.1
		250.0	243.4	2.9	-2.6	247.1	4.1	-1.2	97.6±2.6	98.0±2.8
	Kidney	15.6	14.6	5.1	-6.4	15.2	7.7	-3.0	95.8±6.8	100.0±9.0
		125.0	117.5	3.0	-6.0	118.5	4.9	-5.2	95.1±3.8	99.0±5.8

		250.0	244.8	1.9	-2.1	247.1	4.1	-1.2	99.6±3.8	99.4±5.1
	Stomach	15.6	14.6	5.4	-6.5	15.0	8.7	-4.2	95.4±7.6	97.6±10.5
		125.0	118.6	4.9	-5.1	118.7	5.7	-5.0	95.9±5.8	95.1±6.5
		250.0	243.1	3.1	-2.8	246.4	4.2	-1.5	100.3±3.3	99.2±5.2
	Intestine	15.6	14.6	5.1	-6.4	15.1	7.7	-3.6	100.0±5.8	95.0±7.2
		125.0	118.9	5.3	-4.9	118.9	5.7	-4.9	95.6±5.3	95.8±5.3
		250.0	243.4	2.9	-2.6	246.5	4.4	-1.4	98.7±4.5	100.1±4.6
	Brain	15.6	15.0	9.2	-4.2	15.3	8.6	-2.1	99.0±8.0	92.0±9.2
		125.0	117.5	3.2	-6.0	118.7	5.4	-5.1	95.4±5.7	100.5±5.2
		250.0	244.4	2.2	-2.2	246.7	4.4	-1.3	99.1±4.8	99.8±5.2
Zingerone	Plasma	62.5	63.6	5.9	1.8	64.4	5.1	3.0	103.5±4.3	108.0±4.5
		250.0	244.1	1.8	-2.4	245.6	1.2	-1.8	98.1±1.1	99.3±1.4
		500.0	484.2	2.8	-3.2	487.6	4.0	-2.5	97.7±4.5	100.2±4.8
	Heart	62.5	64.3	4.5	2.8	64.5	4.4	3.2	103.1±4.5	104.2±3.9
		250.0	241. 8	3.5	-3.3	244.0	2.4	-2.4	97.8±2.3	98.0±2.8
		500.0	486.2	3.3	-2.8	489.4	3.8	-2.1	97.1±3.1	101.1±4.0
	Liver	62.5	63.9	5.1	2.3	64.4	4.6	3.0	104.7±4.5	106.2±4.4
		250.0	245.4	2.5	-1.8	244.9	1.9	-2.1	97.2±1.6	97.0±1.9
		500.0	487.5	3.7	-2.5	489.6	4.4	-2.1	97.4±4.8	100.8±5.2
	Spleen	62.5	63.3	6.1	1.2	64.1	5.0	2.6	103.0±5.0	99.8±5.1
		250.0	245.1	1.1	-2.0	246.6	1.5	-1.4	98.9±1.5	109.2±4.7
		500.0	486.6	3.4	-2.7	488.8	4.2	-2.2	98.5±4.2	99.6±1.9
	Lung	62.5	63.4	6.0	1.5	64.2	5.2	2.7	102.1±5.2	100.4±4.5
		250.0	244.4	2.0	-2.2	245.6	1.2	-1.8	97.8±1.2	107.2±5.1
		500.0	486.2	3.3	-2.8	487.7	4.2	-2.5	97.3±3.1	98.4±1.2
	Kidney	62.5	63.3	6.1	1.2	64.5	5.1	3.2	103.2±4.8	101.2±4.0

		250.0	242.4	3.0	-3.0	244.8	1.9	-2.1	98.0±2.0	107.7±5.0
		500.0	480.8	4.8	-3.8	485.8	4.5	-2.8	96.9±4.5	98.6±2.2
	Stomach	62.5	63.1	5.8	0.9	64.1	5.1	2.6	100.9±4.6	101.3±3.7
		250.0	243.2	3.3	-2.7	244.8	2.1	-2.1	99.0±0.5	107.5±4.5
		500.0	482.6	4.4	-3.5	486.0	4.7	-2.8	95.6±3.7	98.4±1.5
	Intestine	62.5	63.9	6.5	2.2	64.4	5.5	3.0	104.5±5.7	97.2±5.2
		250.0	243.0	3.0	-2.8	245.3	1.8	-1.9	97.8±2.0	107.3±5.7
		500.0	480.9	5.1	-3.8	486.5	4.8	-2.7	96.2±4.3	98.7±2.2
	Brain	62.5	63.8	7.0	2.1	64.1	5.9	2.6	102.9±5.6	97.6±5.5
		250.0	240.8	5.0	-3.7	243.9	3.0	-2.5	98.2±1.5	109.1±7.0
		500.0	482.0	5.7	-3.6	486.4	5.3	-2.7	97.7±5.0	97.2±3.5
6-isodehydrogingenone	Plasma	15.6	14.6	5.2	-6.5	14.8	5.2	-5.1	93.5±4.5	95.5±5.8
		125.0	120.3	5.7	-3.8	123.1	4.9	-1.6	98.1±4.6	100.3±5.8
		250.0	242.8	2.6	-2.9	245.1	2.5	-2.0	95.2±4.5	97.2±2.3
	Heart	15.6	14.6	5.2	-6.5	14.8	5.2	-5.1	94.3±3.9	94.4±4.9
		125.0	121.3	4.3	-3.0	123.1	4.3	-1.6	97.1±3.8	98.6±4.8
		250.0	246.2	2.6	-1.5	245.8	2.5	-1.7	97.3±2.1	96.7±2.3
	Liver	15.6	14.9	6.3	-4.4	14.8	6.8	-5.1	94.3±6.2	95.5±7.9
		125.0	121.3	6.6	-3.0	123.7	5.3	-1.0	97.5±5.3	101.1±6.2
		250.0	244.2	3.5	-2.3	246.1	3.0	-1.6	97.5±1.9	96.0±3.0
	Spleen	15.6	14.9	8.7	-4.4	15.1	7.2	-3.6	97.8±7.1	91.5±6.7
		125.0	122.3	2.8	-2.2	122.8	3.8	-1.7	98.5±4.0	98.9±4.7
		250.0	244.5	3.8	-2.2	246.2	3.1	-1.5	98.9±2.9	95.9±2.8
	Lung	15.6	14.3	9.1	-8.7	14.6	7.1	-6.5	91.4±5.9	91.7±5.7
		125.0	121.6	3.8	-2.7	122.4	4.1	-2.1	97.8±3.3	98.2±3.8
		250.0	240.8	3.6	-3.7	243.9	3.0	-2.4	97.1±2.9	96.4±2.8

Kidney	15.6	14.3	8.3	-8.3	14.8	8.6	-5.6	95.9±7.7	94.7±9.8
	125.0	120.8	5.4	-3.4	122.5	4.4	-2.0	98.2±4.4	99.3±5.2
	250.0	243.8	3.2	-2.5	245.5	2.7	-1.8	98.0±2.5	97.0±2.9
Stomach	15.6	14.5	10.8	-7.4	14.5	7.8	-7.0	94.6±7.0	88.1±6.8
	125.0	120.4	6.4	-3.7	123.1	4.8	-1.5	100.2±3.2	100.4±3.8
	250.0	242.6	3.5	-3.0	245.2	2.9	-1.9	99.2±2.5	97.2±3.1
Intestine	15.6	14.7	10.1	-6.0	14.9	7.0	-4.6	97.3±5.8	91.9±6.7
	125.0	119.9	7.4	-4.1	122.7	5.4	-1.8	98.2±5.9	99.5±6.3
	250.0	242.0	3.0	-3.2	244.6	2.6	-2.2	98.0±2.1	97.9±3.0
Brain	15.6	15.1	10.4	-3.4	14.9	8.5	-4.9	94.4±7.1	89.4±3.9
	125.0	120.3	7.2	-3.7	122.8	5.2	-1.8	99.2±4.1	98.4±6.2
	250.0	242.6	4.9	-3.0	244.7	3.3	-2.1	98.3±2.4	96.2±2.5

Table S3: Stability of eight analytes in rat plasma and tissues under various conditions (n = 6).

Analytes	Plasma/tissues	C_{nom} (ng/mL)	4 °C for 24 h		Three freeze-thaw cycles		Frozen at −20°C for 30 days	
			C_{obs} (ng/mL)	R.E. (%)	C_{obs} (ng/mL)	R.E. (%)	C_{obs} (ng/mL)	R.E. (%)
6-gingerol	Plasma	62.5	66.7	6.7	68.1	8.9	68.2	9.2
		250.0	242.7	-2.9	246.1	-1.6	245.9	-1.6
		500.0	490.8	-1.9	485.7	-2.9	484.6	-3.1
	Heart	62.5	67.1	7.3	69.2	10.7	68.8	10.1
		250.0	244.8	-2.1	247.1	-1.2	247.7	-0.9
		500.0	483.1	-3.4	484.7	-3.1	476.1	-4.8
	Liver	62.5	66.0	5.6	68.8	10.0	68.7	10.0
		250.0	244.6	-2.2	248.7	-0.5	248.2	-0.7
		500.0	482.8	-3.5	483.3	-3.3	484.3	-3.1
	Spleen	62.5	66.4	6.2	67.4	7.9	67.9	8.7
		250.0	246.0	-1.6	249.3	-0.3	248.9	-0.5
		500.0	474.7	-5.1	478.3	-4.4	476.1	-4.8
	Lung	62.5	65.7	5.1	67.0	7.2	66.6	6.6
		250.0	244.3	-2.3	247.3	-1.1	246.4	-1.4
		500.0	487.3	-2.5	490.3	-1.9	485.2	-3.0
	Kidney	62.5	66.9	7.0	69.5	11.2	69.5	11.2
		250.0	247.7	-0.9	249.8	-0.1	248.3	-0.7
		500.0	491.5	-1.7	487.1	-2.6	491.0	-1.8
	Stomach	62.5	67.0	7.1	69.0	10.4	68.7	9.8
		250.0	247.1	-1.2	247.3	-1.1	246.7	-1.3
		500.0	473.6	-5.3	476.1	-4.8	474.5	-5.1
	Intestine	62.5	67.5	8.0	69.5	11.3	68.8	10.1

		250.0	245.4	-1.9	250.0	0.0	249.6	-0.2
		500.0	483.6	-3.3	477.5	-4.5	475.4	-4.9
	Brain	62.5	67.4	7.8	69.4	11.1	69.4	11.1
		250.0	244.5	-2.2	247.9	-0.8	248.6	-0.6
		500.0	490.4	-1.9	488.0	-2.4	492.0	-1.6
6-shogaol	Plasma	62.5	62.9	0.7	64.0	2.4	64.2	2.7
		250.0	244.5	-2.2	250.1	0.0	250.6	0.2
		500.0	485.4	-2.9	485.7	-2.9	484.0	-3.2
	Heart	62.5	62.7	0.3	63.8	2.1	63.5	1.6
		250.0	245.4	-1.8	249.8	-0.1	249.8	-0.1
		500.0	480.6	-3.9	476.1	-4.8	478.3	-4.3
	Liver	62.5	63.2	1.1	64.7	3.5	64.3	2.9
		250.0	247.8	-0.9	253.4	1.4	254.5	1.8
		500.0	488.2	-2.4	485.3	-2.9	488.5	-2.3
	Spleen	62.5	63.2	1.1	64.8	3.7	65.4	4.6
		250.0	248.2	-0.7	254.0	1.6	256.0	2.4
		500.0	491.3	-1.8	491.9	-1.6	498.7	-0.3
	Lung	62.5	64.9	3.8	64.3	2.8	64.1	2.6
		250.0	249.6	-0.2	250.3	0.1	249.9	-0.0
		500.0	483.1	-3.4	483.3	-3.3	483.8	-3.2
	Kidney	62.5	63.6	1.8	64.1	2.6	64.5	3.2
		250.0	245.8	-1.7	252.1	0.8	252.9	1.2
		500.0	487.3	-2.5	485.6	-2.9	481.7	-3.7
	Stomach	62.5	63.3	1.3	63.9	2.2	64.0	2.4
		250.0	249.5	-0.2	249.8	-0.1	250.0	0.0
		500.0	483.2	-3.4	487.4	-2.5	485.1	-3.0

	Intestine	62.5	63.7	1.9	64.9	3.8	65.5	4.7
		250.0	249.9	-0.0	255.7	2.3	257.7	3.1
		500.0	491.7	-1.7	490.9	-1.8	499.8	-0.0
	Brain	62.5	63.2	1.1	63.8	2.1	64.5	3.2
		250.0	248.6	-0.6	252.0	0.8	252.9	1.2
		500.0	485.7	-2.9	484.3	-3.1	482.1	-3.6
8-gingerol	Plasma	62.5	60.9	-2.5	61.9	-1.0	62.1	-0.7
		250.0	254.2	1.7	257.0	2.8	256.7	2.7
		500.0	485.6	-2.9	483.6	-3.3	482.9	-3.4
	Heart	62.5	60.4	-3.3	62.4	-0.2	61.9	-0.9
		250.0	256.8	2.7	261.3	4.5	260.9	4.4
		500.0	478.1	-4.4	484.7	-3.1	476.3	-4.7
	Liver	62.5	61.9	-1.0	63.6	1.7	63.6	1.7
		250.0	258.6	3.4	261.6	4.6	260.1	4.0
		500.0	497.0	-0.6	500.1	0.0	495.1	-1.0
	Spleen	62.5	61.9	-1.0	64.0	2.4	63.8	2.1
		250.0	257.9	3.2	261.5	4.6	262.3	4.9
		500.0	487.4	-2.5	489.8	-2.1	488.1	-2.4
	Lung	62.5	60.7	-2.9	62.2	-0.5	61.9	-1.0
		250.0	255.6	2.3	258.6	3.4	258.3	3.3
		500.0	484.9	-3.0	492.3	-1.5	486.6	-2.7
	Kidney	62.5	60.5	-3.3	61.9	-1.0	62.5	-0.0
		250.0	254.1	1.6	256.7	2.7	258.3	3.3
		500.0	490.3	-1.9	489.8	-2.0	496.4	-0.7
	Stomach	62.5	61.8	-1.1	62.5	0.1	62.3	-0.4
		250.0	257.6	3.0	258.0	3.2	257.7	3.1

		500.0	482.8	-3.4	486.9	-2.6	486.2	-2.8
	Intestine	62.5	62.1	-0.7	63.7	1.9	63.5	1.6
		250.0	255.9	2.3	260.0	4.0	259.1	3.6
		500.0	491.9	-1.6	486.9	-2.6	487.0	-2.6
	Brain	62.5	60.6	-3.0	62.5	0.1	62.6	0.1
		250.0	254.7	1.9	258.0	3.2	258.1	3.3
		500.0	489.3	-2.2	486.4	-2.7	493.3	-1.3
8-shogaol	Plasma	15.6	15.7	0.2	15.4	-1.3	15.6	0.0
		125.0	118.0	-5.6	120.6	-3.5	120.5	-3.6
		250.0	257.5	3.0	257.6	3.1	257.6	3.0
	Heart	15.6	15.4	-1.7	15.7	0.2	15.4	-1.7
		125.0	120.9	-3.3	122.3	-2.2	121.6	-2.7
		250.0	255.6	2.2	258.2	3.3	257.7	3.1
	Liver	15.6	15.7	0.5	15.8	1.1	16.3	4.4
		125.0	125.7	0.6	125.1	0.0	122.9	-1.7
		250.0	254.7	1.9	258.2	3.3	257.7	3.1
	Spleen	15.6	15.2	-2.7	15.0	-4.1	14.6	-6.7
		125.0	123.4	-1.3	127.5	2.0	127.7	2.2
		250.0	260.1	4.0	260.8	4.3	264.8	5.9
	Lung	15.6	15.8	0.8	15.8	0.9	15.8	1.1
		125.0	121.5	-2.8	121.8	-2.6	121.7	-2.7
		250.0	257.8	3.1	256.8	2.7	257.3	2.9
	Kidney	15.6	16.1	3.1	15.9	1.7	15.9	1.6
		125.0	121.2	-3.1	121.4	-2.9	122.2	-2.3
		250.0	258.4	3.4	258.4	3.4	259.1	3.6
	Stomach	15.6	15.3	-2.1	15.6	-0.4	15.4	-1.2

		125.0	124.0	-0.8	123.3	-1.4	123.9	-0.9
		250.0	256.6	2.6	256.5	2.6	256.8	2.7
	Intestine	15.6	15.2	-2.8	14.8	-5.5	14.4	-8.1
		125.0	123.4	-1.3	125.9	0.7	127.7	2.2
		250.0	260.7	4.3	260.6	4.2	264.7	5.9
	Brain	15.6	16.1	3.3	15.7	0.5	15.6	-0.3
		125.0	118.4	-5.3	121.4	-2.9	122.2	-2.3
		250.0	258.8	3.5	258.2	3.3	258.3	3.3
10-gingerol	Plasma	62.5	65.0	4.0	66.5	6.4	66.8	6.8
		250.0	248.4	-0.6	255.1	2.0	256.9	2.8
		500.0	496.9	-0.6	502.8	0.6	498.0	-0.4
	Heart	62.5	63.7	1.9	64.4	3.0	65.9	5.5
		250.0	252.2	0.9	256.5	2.6	255.1	2.0
		500.0	500.5	0.1	500.6	0.1	494.0	-1.2
	Liver	62.5	65.2	4.4	65.6	5.0	66.0	5.5
		250.0	249.8	-0.1	251.7	0.7	249.3	-0.3
		500.0	509.0	1.8	509.3	1.9	508.1	1.6
	Spleen	62.5	63.3	1.3	65.5	4.8	65.1	4.2
		250.0	252.3	0.9	260.7	4.3	264.4	5.8
		500.0	507.4	1.5	515.1	3.0	513.1	2.6
	Lung	62.5	64.2	2.7	65.9	5.4	65.4	4.7
		250.0	254.1	1.7	258.8	3.5	258.2	3.3
		500.0	487.7	-2.5	483.4	-3.3	480.1	-4.0
	Kidney	62.5	65.6	5.0	66.9	7.0	66.4	6.3
		250.0	250.2	0.1	255.7	2.3	258.1	3.2
		500.0	506.1	1.2	512.0	2.4	512.2	2.4

	Stomach	62.5	66.5	6.4	66.9	7.0	66.8	6.9
		250.0	255.1	2.0	256.5	2.6	258.7	3.5
		500.0	508.0	1.6	510.2	2.0	505.5	1.1
	Intestine	62.5	65.4	4.7	67.1	7.3	66.7	6.7
		250.0	254.3	1.7	260.4	4.2	264.4	5.8
		500.0	509.2	1.8	515.1	3.0	513.1	2.6
	Brain	62.5	64.3	2.9	66.6	6.5	66.2	6.0
		250.0	250.0	0.0	256.3	2.5	259.0	3.6
		500.0	503.8	0.8	511.5	2.3	512.1	2.4
10-shogaol	Plasma	15.6	14.7	-6.0	15.0	-4.1	14.6	-6.5
		125.0	116.8	-6.6	120.1	-3.9	120.9	-3.3
		250.0	242.8	-2.9	246.3	-1.5	248.3	-0.7
	Heart	15.6	15.2	-3.1	15.9	2.0	15.9	2.0
		125.0	119.4	-4.5	123.5	-1.2	125.5	0.4
		250.0	247.5	-1.0	252.6	1.0	253.2	1.3
	Liver	15.6	15.4	-1.7	16.0	2.1	16.2	3.4
		125.0	119.8	-4.2	122.9	-1.7	123.0	-1.6
		250.0	245.0	-2.0	248.7	-0.5	247.3	-1.1
	Spleen	15.6	14.9	-4.4	15.1	-3.3	15.0	-4.0
		125.0	119.2	-4.7	123.3	-1.4	122.8	-1.7
		250.0	247.2	-1.1	248.9	-0.5	251.2	0.5
	Lung	15.6	14.6	-6.5	14.9	-4.5	15.0	-4.3
		125.0	116.9	-6.5	119.8	-4.2	120.2	-3.9
		250.0	244.5	-2.2	246.7	-1.3	247.3	-1.1
	Kidney	15.6	15.1	-3.5	14.9	-4.9	14.8	-5.5
		125.0	119.5	-4.4	121.3	-2.9	121.3	-3.0

		250.0	244.8	-2.1	248.5	-0.6	250.2	0.1
	Stomach	15.6	14.9	-4.4	14.7	-5.9	14.3	-8.3
		125.0	122.4	-2.1	121.2	-3.1	121.4	-2.9
		250.0	248.2	-0.7	247.1	-1.2	249.4	-0.2
	Intestine	15.6	15.6	-0.5	15.2	-2.9	14.6	-6.3
		125.0	120.0	-4.0	123.1	-1.5	122.6	-1.9
		250.0	249.8	-0.1	250.8	0.3	253.6	1.5
	Brain	15.6	15.3	-2.2	15.3	-2.1	14.8	-5.1
		125.0	118.5	-5.2	121.6	-2.7	121.5	-2.8
		250.0	244.7	-2.1	247.9	-0.9	250.0	0.0
Zingerone	Plasma	62.5	63.8	2.1	63.7	1.9	63.5	1.6
		250.0	243.1	-2.8	244.1	-2.4	244.3	-2.3
		500.0	482.5	-3.5	479.3	-4.1	478.8	-4.3
	Heart	62.5	63.5	1.5	64.4	3.0	64.2	2.7
		250.0	243.3	-2.7	245.0	-2.0	242.6	-3.0
		500.0	486.9	-2.6	484.6	-3.1	484.4	-3.1
	Liver	62.5	64.0	2.4	64.4	3.0	64.2	2.7
		250.0	243.8	-2.5	245.0	-2.0	245.9	-1.6
		500.0	482.4	-3.5	482.3	-3.5	484.5	-3.1
	Spleen	62.5	63.8	2.1	62.9	0.6	62.0	-0.8
		250.0	247.4	-1.1	247.7	-0.9	248.4	-0.7
		500.0	492.4	-1.5	488.5	-2.3	495.6	-0.9
	Lung	62.5	63.1	1.0	63.6	1.8	63.4	1.4
		250.0	246.4	-1.5	245.4	-1.8	244.2	-2.3
		500.0	482.1	-3.6	476.6	-4.7	478.1	-4.4
	Kidney	62.5	64.2	2.7	64.5	3.2	64.5	3.2

		250.0	244.9	-2.0	246.2	-1.5	247.1	-1.1
		500.0	484.6	-3.1	479.7	-4.1	476.4	-4.7
	Stomach	62.5	63.1	1.0	63.5	1.6	63.1	0.9
		250.0	243.0	-2.8	243.9	-2.5	243.2	-2.7
		500.0	484.7	-3.1	483.4	-3.3	480.0	-4.0
	Intestine	62.5	62.6	0.1	62.6	0.1	61.7	-1.3
		250.0	245.5	-1.8	247.0	-1.2	247.7	-0.9
		500.0	497.8	-0.4	489.5	-2.1	496.6	-0.7
	Brain	62.5	64.7	3.6	63.9	2.2	63.9	2.2
		250.0	243.7	-2.5	245.6	-1.8	247.1	-1.1
		500.0	486.6	-2.7	479.0	-4.2	474.0	-5.2
6-isodehydrogingenone	Plasma	15.6	14.1	0.7	14.7	-5.9	14.9	-4.9
		125.0	120.2	-2.2	120.8	-3.3	120.1	-4.0
		250.0	243.8	-2.9	246.6	-1.4	245.9	-1.6
	Heart	15.6	14.8	0.3	15.2	-2.7	15.0	-3.8
		125.0	122.8	-1.8	124.8	-0.1	122.6	-1.9
		250.0	244.7	-3.9	248.5	-0.6	247.6	-1.0
	Liver	15.6	14.6	1.1	15.1	-3.7	14.9	-4.8
		125.0	126.4	-0.9	127.0	1.6	125.0	0.0
		250.0	246.1	-2.4	248.7	-0.5	249.8	-0.1
	Spleen	15.6	14.5	1.1	15.5	-0.8	15.5	-1.2
		125.0	121.5	-0.7	120.8	-3.3	121.1	-3.1
		250.0	246.8	-1.8	249.0	-0.4	248.5	-0.6
	Lung	15.6	14.5	3.8	14.9	-4.8	14.7	-6.0
		125.0	120.3	-0.2	121.2	-3.1	120.7	-3.4
		250.0	243.2	-3.4	245.8	-1.7	244.9	-2.0

Kidney	15.6	14.4	1.8	14.8	-5.3	15.0	-4.2
	125.0	125.2	-1.7	124.2	-0.6	125.5	0.4
	250.0	245.0	-2.5	248.4	-0.7	248.6	-0.6
Stomach	15.6	14.6	1.3	15.0	-3.7	15.0	-3.8
	125.0	120.6	-0.2	120.0	-4.0	118.8	-5.0
	250.0	246.8	-3.4	247.8	-0.9	247.2	-1.1
Intestine	15.6	14.9	1.9	15.4	-1.7	15.3	-2.0
	125.0	123.9	-0.0	122.8	-1.7	123.1	-1.5
	250.0	247.4	-1.7	248.9	-0.4	248.8	-0.5
Brain	15.6	14.7	1.1	15.1	-3.2	15.3	-2.1
	125.0	124.8	-0.6	125.1	0.1	127.1	1.7
	250.0	243.8	-2.9	247.0	-1.2	247.2	-1.1
